# Few-shot biomedical NER empowered by LLMs-assisted data augmentation and multi-scale feature extraction

**DOI:** 10.1186/s13040-025-00443-y

**Published:** 2025-04-04

**Authors:** Di Zhao, Wenxuan Mu, Xiangxing Jia, Shuang Liu, Yonghe Chu, Jiana Meng, Hongfei Lin

**Affiliations:** 1 School of Computer Science and Engineering, Dalian Minzu University, Jinshitan Street, Jinzhou District, Dalian, 116650 Liaoning China; 2 School of Computer Science and Technology, Dalian University of Technology, Dalian, 116024 Liaoning China; 3 Postdoctoral Workstation of Dalian Yongia Electronic Technology Co., Ltd, Dalian, 116024 Liaoning China; 4 Nantong University, Nantong, 226019 Jiangsu China

**Keywords:** Few-shot learning, ChatGPT, Data augmentation, Named entity recognition

## Abstract

Named Entity Recognition (NER) is a fundamental task in processing biomedical text. Due to the limited availability of labeled data, researchers have investigated few-shot learning methods to tackle this challenge. However, replicating the performance of fully supervised methods remains difficult in few-shot scenarios. This paper addresses two main issues. In terms of data augmentation, existing methods primarily focus on replacing content in the original text, which can potentially distort the semantics. Furthermore, current approaches often neglect sentence features at multiple scales. To overcome these challenges, we utilize ChatGPT to generate enriched data with distinct semantics for the same entities, thereby reducing noisy data. Simultaneously, we employ dynamic convolution to capture multi-scale semantic information in sentences and enhance feature representation based on PubMedBERT. We evaluated the experiments on four biomedical NER datasets (BC5CDR-Disease, NCBI, BioNLP11EPI, BioNLP13GE), and the results exceeded the current state-of-the-art models in most few-shot scenarios, including mainstream large language models like ChatGPT. The results confirm the effectiveness of the proposed method in data augmentation and model generalization.

## Introduction

Named Entity Recognition (NER) is a foundational task in the domain of Natural Language Processing (NLP), with its performance significantly influencing various related endeavors. Particularly in the biomedical field. In recent years, deep learning-based supervised NER methodologies [1–3] have developed rapidly, achieving highly competitive results. However, these achievements are highly dependent on the support of large amounts of data.

In the biomedical field, obtaining annotated data is extremely challenging. This process requires not only numerous experts but also considerable time. Even some large labeled datasets, such as electronic case data, are withheld from public access due to privacy and security concerns. Therefore, it is urgent to improve the effectiveness of the model in few-shot scenarios.

Few-shot Learning (FSL) [4–6] is a machine learning method that requires only a small amount of training data. Given that many biomedical datasets have limited training samples in real-world applications, FSL-based methods hold significant potential for biomedical NER tasks.

Currently, there are two main approaches to NER based on FSL. The first approach involves researchers designing new models. For example, [7] proposed a model that combines meta-learning and transfer learning for few-shot NER, demonstrating strong performance on a 20-shot task. Huang et al. [8] achieved state-of-the-art (SOTA) results in few-shot and zero-shot scenarios by employing techniques such as supervised pre-training and self-training. Additionally, Das et al. [9] addressed overfitting issues arising from source domain training by using Contrastive Learning (CL) to optimize the distribution distance of embeddings for cross-labeled entities. He et al. [10] achieved CL in few-shot scenario by initializing semantic anchors and employing template-free prompts. The prompts, along with the input embeddings, were contextually optimized using the proposed semantic-enhanced CL loss. The other approach involves Data Augmentation (DA) strategies, often using methods like data shifting, reversing, or entity replacement. Chen et al. [11] proposed cross-domain DA for NER tasks, converting data representations from high-resource domains to low-resource domains by learning patterns (such as styles, noises, abbreviations, etc.), achieving significant results in few-shot NER tasks. However, traditional DA methods still have certain limitations, such as shortcomings in generating sentence samples that are natural, fluent, and comply with grammar rules.

Therefore, this paper proposes a multi-scale feature extraction model assisted by a large language model (LLMs). First, we use ChatGPT with prompt techniques to perform DA on the original data, generating semantically similar new sentences. Additionally, multi-scale feature fusion is used to enhance feature learning from multiple perspectives, enabling the model to better understand the text. Unlike KGPC [12], which relies on structured knowledge graphs for entity replacement, our approach leverages ChatGPT to generate contextually diverse sentences while preserving semantic consistency. This not only avoids the rigidity of predefined semantic relations but also captures natural language variations crucial for real-world biomedical texts. Furthermore, our multi-scale feature extraction module addresses the limitation of transformer-based models in local pattern recognition, enabling finer-grained entity boundary detection compared to KGPC’s QA-style prompt learning.

Our experiments show that the data generated by ChatGPT is more coherent and aligns better with the original meaning. When used for training, this high-quality data generates less noise, which is particularly important in few-shot scenarios. In addition, the multi-scale feature fusion approach enhances the model’s capacity to capture both fine-grained and global contextual information, effectively addressing BERT’s limitations in handling short-range dependencies (e.g., compound words and abbreviations). For instance, in Biomedical NLP tasks such as recognizing gene names like “IL-2 receptor”, our method simultaneously leverages character-level features (“IL-2”) and word-level features (“receptor”). Furthermore, compared to static convolutional methods, our approach adaptively captures local patterns of varying lengths, particularly beneficial for handling biomedical term variations (e.g., “EGFR” vs. “epidermal growth factor receptor”), thereby significantly improving model robustness and accuracy. The source code and datasets supporting this research are publicly available at https://github.com/MWXGOD/ChatGPTNER. Our key contributions are as follows:

- We employed ChatGPT for DA, mitigating the issues of decreased semantic naturalness and diversity caused by traditional methods such as random replacement and deletion.
- We introduced a multi-scale feature extraction module to alleviate the issue of insufficient local feature extraction in traditional transformer-based pre-trained models.
- Our approach achieved the best performance in most few-shot scenarios across three out of four datasets. In the BC5CDR-Disease dataset, compared to the previous state-of-the-art (SOTA) model, our method increased the F1 scores by 10.2%, 14.4%, and 15.2% in the 5-shot, 20-shot, and 50-shot scenarios, respectively.

## Related work

### NER

NER is a crucial task in NLP, particularly within the biomedical domain. In recent years, supervised NER methods have made significant progress with the rapid development of deep learning. However, these methods usually require large amounts of labeled data for model fine-tuning. In the biomedical field, labeled data is often limited, and some large-scale labeled datasets are not publicly available due to privacy or security issues. This limitation restricts the application of supervised learning methods.

The early NER methods are mainly categorized into sequence labeling methods and span-based methods. The former considers the NER task as a sequence labeling problem, specifically, predefining an annotation scheme (e.g., BIO, BIOES, etc.), and then the model assigns a label to each token in the sentence. The latter enumerates all possible spans in the sentence and determines whether each span is a complete entity mention and its category, i.e., predicts the start and end positions of the entities corresponding to each category.

The sequence labeling method transforms the NER task into a classification task for each token in the sentence, making an excellent decoding structure critical. Early models, such as Convolutional Neural Network (CNN) [13–15] and CRF [16, 17], and recent models, such as LSTM [13, 16] and Transformer [18, 19], have been employed for this purpose. However, these sequence labeling methods rely heavily on predefined entity types and large-scale datasets, making it difficult to generalize effectively in new label or few-shot scenarios.

Some span-based methods utilize the prompt technique [20–22], posing questions related to the original sentence. Specifically, the input sentence is converted into a question like “Is span1 a label1 entity in the input sentence?” to identify the specific span and entity type. Another successful approach is to address the NER task using a pipeline [23, 24]: first for span detection, then for span classification. The drawback of these span-based approaches is the need to consider all possible entity spans, resulting in an exponential growth of the search space. This can cause significant computational overhead when processing long texts.

### Few-shot learning

NER tasks often lack sufficient training data in real-world scenarios, making the application of FSL increasingly popular. Few-shot NER uses a small amount of annotated data to train the model, enhancing its generalization ability. Its main methods include metric learning, prototype networks, DA and meta-learning.

Metric learning aims to create a metric space where similar samples are closer together and dissimilar samples are farther apart. This enables the model to better distinguish between different entity types, even with scarce training samples. The prototype network [25, 26] is a classification method that classifies new samples by learning prototype vectors for each category. In FSL, prototype networks construct prototypes for each category from a small number of samples and are used to recognize new named entities.

Meta-learning [27, 28] is a technique that enhances a model’s generalization ability by rapidly learning from a limited number of tasks. In FSL, meta-learning assists models in adapting to new categories.

DA [29] is a technique that expands the training data by transforming the training samples. In FSL, DA can improve the model’s adaptability to different samples and make it more generalizable.

In recent years, DA has garnered significant attention in few-shot NER. Traditional DA methods (e.g., entity replacement, random deletion, random insertion) have limitations, especially in the biomedical domain. Since textual information in the biomedical domain is highly objective and logical, it is easy to cause semantic and syntactic errors in the text when using traditional DA methods. Therefore, we employ LLMs (such as ChatGPT) for DA, significantly reducing semantic and syntactic errors in the generated text.

## Methods

**Fig. 1:**
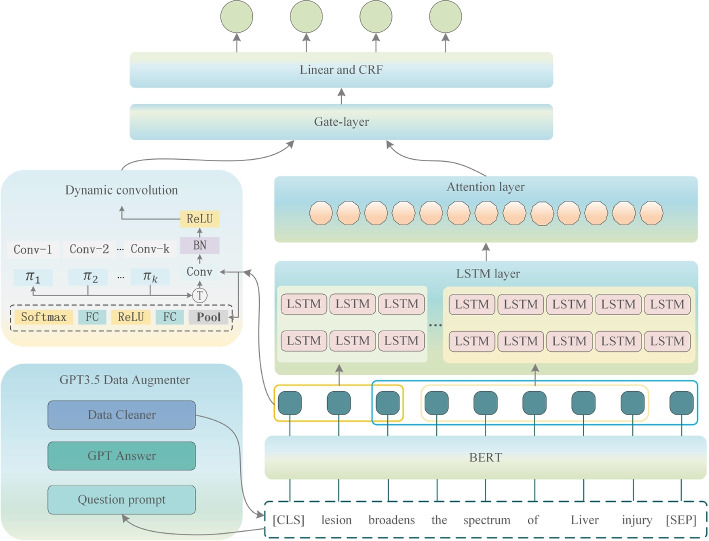
Model framework diagram: It includes data augmentaion, encoder, multi-scale feature extraction, dynamic convolution, attention, and gating mechanisms

In this section, we formally describe the structure of the presented model, as illustrated in Fig. 1.

Our overall model design can be broadly divided into four components: the Data augmentation section, the encoder section, the Multi-scale feature extraction section, and the Feature fusion section. Below is a detailed description of each component of the model:

### Data augmentation

**Fig. 2:**
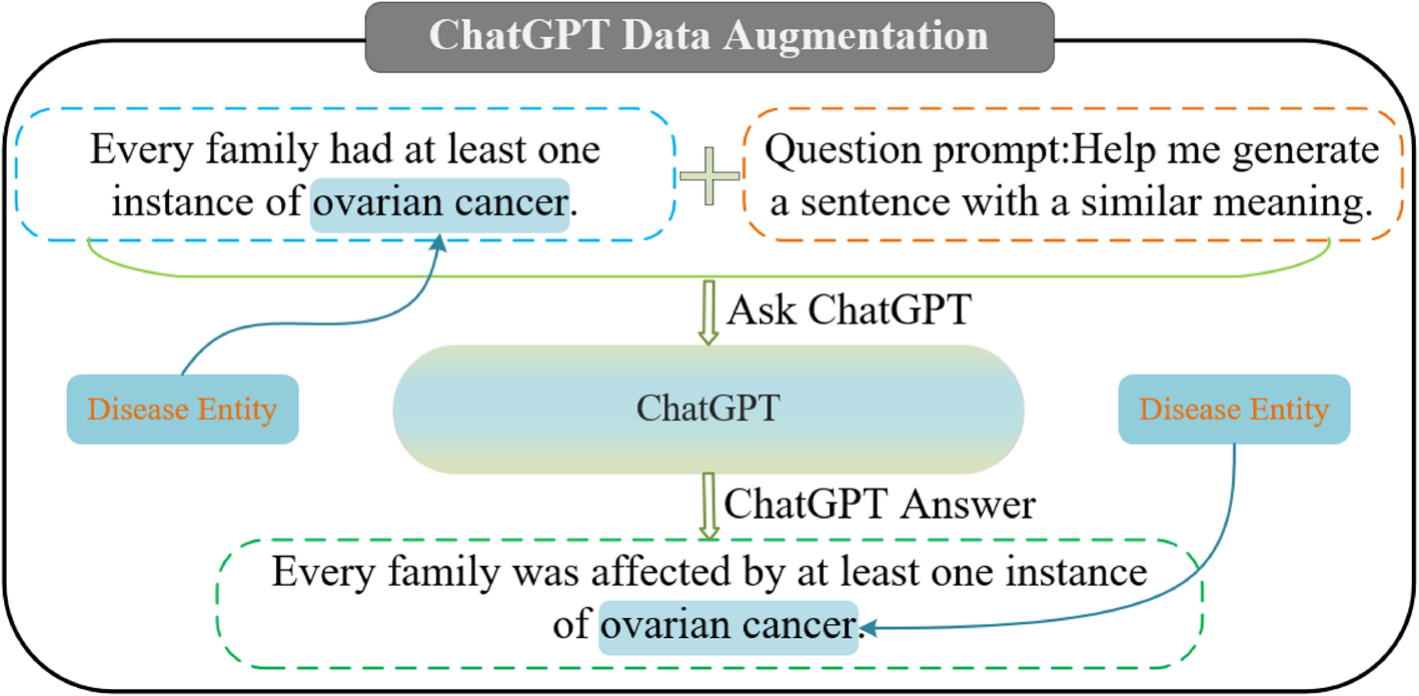
Formulate questions for ChatGPT using original sentence splicing templates to generate multiple sets of augmented data

To address the challenges of few-shot NER tasks, DA techniques are extensively applied to reduce the need for manual annotation and introduce diverse examples, thereby enhancing the model’s generalization capability. In this paper, we use the popular model ChatGPT as an auxiliary tool to generate synthetic data closely resembling the original samples. The specific procedure is as follows:

As shown in Fig. 2, we first design a query template: “Original sentence + Help me rephrase this sentence while preserving the original meaning.” Using this template, we repeat the inquiry to ChatGPT five times, expanding the original dataset fivefold. Subsequently, we perform entity localization to ensure the generated data includes entity consistent with the original samples. Finally, after data generation, we meticulously filter and clean the data to eliminate ineffective entries that cannot precisely locate entities, ensuring only high-quality, meaningful augmented data remains.

This meticulous data filtering process contributes to dataset expansion and enhances the model’s generalization performance. By fully utilizing the ChatGPT model and conducting rigorous data selection, we provide a larger and more diverse training dataset for few-shot NER tasks, thereby further enhancing the model’s performance.

### Encoder

First, define the input text sequence $X=(x_1,x_2,...x_i)$, where $x_i$ represents the i-th word in the text sequence, and *i* represents the number of token contained in the text sequence.

Given the specificity of biomedical text and the model’s performance under low-resource conditions, we have chosen PubMedBERT as our encoder component. Thanks to PubMedBERT’s advantage of being pretrained on a substantial corpus of biomedical literature, it offers our model a superior ability to comprehend context. We feed the processed text sequence *X* into the PubMedBERT model for encoding, resulting in a feature vector representation matrix for this text sequence:

1 $$\begin{aligned} E={e_1,e_2,...e_i}=BERT(X) \end{aligned}$$

### Multi-scale feature extraction

Models based on Transformer structures like BERT benefit from the multi-head attention mechanism, which excels at capturing long-range contextual features. However, this can lead to the neglect of some local features. To address this, we further split the features *E* extracted from BERT into multiple scales, following different lengths $K={k_1,k_2,...k_j}$, resulting in sub-features $L_{k_{1}},L_{k_{2}},...L_{k_{j}}$, where $k_1$,$k_2$ represent different lengths of scales, and j represents the number of different scales. $L_{k_{1}}$ represents the features encoded by PubMedBERT at scale $k_1$, and $L_{k_{2}}$,$L_{k_{j}}$ are similar.

Next, we use BiLSTM to perform further local feature extraction on the local features $L_{k_{1}},L_{k_{2}},...,L_{k_{j}}$, obtaining features of different scales for the sentences: $H_{k_{1}},H_{k_{2}},...H_{k_{j}}$. We set the same BiLSTM output dimension for consistency, so $H_{k_{1}},H_{k_{2}},...H_{k_{j}}$ have the same dimensions. We concatenate these features to form *H*, which we will use later.

2 $$\begin{aligned} H={H_{k_{1}},H_{k_{2}},...H_{k_{j}}} = BiLSTM(L_{k_{1}},L_{k_{2}},...L_{k_{j}}) \end{aligned}$$

To further refine features, we introduce the dynamic CNN, a technique commonly used in the visual domain. In the visual domain, convolution typically applies to multi-dimensional features, such as 3D for images or higher dimensions. However, for text, we use 1D convolution. Here, we adapt dynamic CNN to 1D convolution to extract local features from text.

Applying dynamic convolution in the text domain presents several advantages. First, biomedical text contains many nonlinear features, including syntactic structures and semantic relationships. Dynamic convolution introduces nonlinear transformations that capture these features more effectively, enhancing text processing performance and model generalization. Second, dynamic convolution enables feature extraction at various scales, from character-level to word-level, and even larger structures, enhancing multi-scale adaptability in text processing.

Previously, we used PubMedBERT to extract sentence features *E*. Now, we feed *E* into the dynamic convolution module, resulting in local sentence features $D = {d_1, d_2, \ldots , d_i}$, as shown in Formula (3):

3 $$\begin{aligned} D = Dconv(E) \end{aligned}$$

### Feature fusion

In the previous sections, we extracted sentence features *E* from PubMedBERT and multi-scale local features *H* using BiLSTM. Now, we merge these features using an attention mechanism. We employ a linear layer to concatenate the local features with the global features, as shown in formula (4):

4 $$\begin{aligned} o_l = Linear(E,H) \end{aligned}$$

Subsequently, we use $o_l$ as *Q*, with *E* and *H* serving as key-value matrices. The final attention output is calculated as shown in formula (5):

5 $$\begin{aligned} O=softmax(o_l [E,H_a ]^T)[E,H] \end{aligned}$$

To effectively combine the output from the dynamic convolution module with the output in (5) and enhance the stability of text features, we employ a gating mechanism to dynamically blend vectors from both modules. This results in the final vector representation, as shown in formulas (6) and (7):

6 $$\begin{aligned} g= sigmoid(W_o O+ b_o + W_{dconv} D + b_{dconv}) \end{aligned}$$

7 $$\begin{aligned} h = g \odot O + (1-g) \odot D \end{aligned}$$

In these formulas, *O* represents the attention output from formula (5), and *D* is the output from the dynamic convolution. $W_o, W_{dconv}, b_o,$ and $b_{dconv}$ are learnable parameters. Finally, *h* is fed into a Linear layer and a CRF layer for entity type prediction, obtaining $\hat{y}$ as shown in formula (8):

8 $$\begin{aligned} \hat{y} = CRF(Linear(h)) \end{aligned}$$

## Experiments

In this section, we provide a detailed overview of our experiments, including data, low-resource settings, comparison models, main results, and analysis.

### Data

To evaluate our model’s performance in low-resource settings, we tested it on the NCBI, BC5CDR-Disease, BioNLP11EPI, and BioNLP13GE datasets. These datasets include: The NCBI dataset [30] is derived from NER and concept normalization, comprising 793 PubMed abstracts annotated for disease entities. The BC5CDR-Disease dataset [31] includes 1,500 annotated PubMed articles focused on disease mentions, divided into three sets of 500 articles each for training, development, and testing. Additionally, the BioNLP dataset, which includes BioNLP11EPI [32] and BioNLP13GE [33], comes from Biomedical Natural Language Processing Workshops and covers fundamental entities such as genes and proteins. Notably, all these datasets adhere to BIO tagging conventions.

### Low-resource setting

To simulate low-resource scenarios, we used random sampling to subsample sentences from the original dataset, creating K-shot support sets for the four datasets. We randomly sampled 5, 20, and 50 sentences for each shot. For each shot, we performed five random samplings with different random number seeds, resulting in five training data sets in the final low-resource training set. For the validation set, we did not use the entire validation set. Instead, we partitioned the training and validation sets in an 8:2 ratio for each shot. Model evaluation was conducted on the complete test set using the mean F1 score with standard deviation as the evaluation metric.

We used PubMedBERT as the encoder with a hidden layer size of 256 and a dropout rate of 0.5. For the BiLSTM, the hidden layer size was set to 256, with scale quantities of 3, 5, and 7. The dynamic convolution kernel size was set to 5 with a padding of 2, and the learning rate was configured to 1e-5. The experiments were conducted on a single RTX 3090 GPU.

### Compared models

In our experiment, we compare our approach against several contemporary SOTA FSL models in the context of BioNER task.

TransferBERT [34] is a domain transfer model for sequence labeling. It is first pre-fine-tuned on high-resource domains and then further fine-tuned on low-resource domains.

DaGa [29] proposed a novel method by synthesizing high-quality data through language models trained on linearized labeled sentences. This approach effectively enhances performance in low-resource tagging tasks. Authors conducted extensive experiments in both supervised and semi-supervised settings, validating the method’s efficacy, particularly showcasing remarkable performance in scenarios with scarce training data.

NNshot [35] utilizes a supervised NER model trained in the source domain as a feature extractor, demonstrating the superior effectiveness of nearest neighbor classifiers in the feature space compared to standard meta-learning methods. Moreover, it introduces a cost-effective approach to capture dependencies among entity labels.

LightNER [36] addresses low-resource NER problems by introducing a method tailored for such scenarios. It employs a learnable entity category tokenizer and a pluggable guiding module to tackle challenges arising from diverse label sets and scarce data. This approach renders LightNER flexible and highly effective in low-resource contexts.

FFF-NER [37] introduces a novel few-shot NER fine-tuning framework that adopts a new labeling approach. It formalizes NER fine-tuning into label prediction or generation based on the choice of pre-trained models.

Zhou et al. [38] introduces a novel framework called Masked Entity Language Modeling (MELM). This framework injects NER labels into the context of sentences to predict masked entity tags under explicit conditions. It generates high-quality augmented data encompassing new entities, thereby offering rich knowledge about entity patterns and significantly enhancing NER performance.

KGPC [12] introduces a knowledge-guided instance generation method utilizing domain knowledge graphs to generate diverse and novel entities based on similar semantic relations of neighboring nodes. Additionally, by incorporating question prompts and prompt CL, KGPC frames BioNER as a question-answering task to enhance model performance.

In addition, we compared the results of four advanced LLMs. Among them, Google developed Gemini-1.0 and Gemini-1.5, while OpenAI developed GPT-3.5-turbo and GPT-3.5-turbo-0125.

### Main result

Table 1 presents our experimental results, comparing our method with seven models and four LLMs. Notably, our approach achieved the best performance in majority-shot instances across three out of four datasets. The substantial improvement in the BC5CDR-Disease dataset was particularly striking, with F1 score gains of 10.2%, 9.4%, and 9.2% in 5-shot, 20-shot, and 50-shot scenarios, respectively, compared to the previous SOTA model. In the BioNLP13GE dataset, our method showed improvements over the eleven baseline models in all shot sizes, except the 5-shot scenario.

**Table 1:** Model performance (F1 scores (%)) on NCBI, BC5CDR-Disease, BioNLP11EPI, and BioNLP13GE datasets

Dataset	Model	5-shot	20-shot	50-shot
NCBI	TransferBERT(2019) [34]	31.3±7.0	50.5±2.9	54.3±3.9
	Daga(2020) [29]	25.2±7.6	47.0±6.6	56.0±2.8
	NNshot(2020) [35]	23.1±3.7	33.1±5.6	37.5±3.1
	LightNER(2022) [36]	47.0±5.1	58.1±2.3	59.5±4.0
	FFF-NER(2022) [37]	32.7±8.5	49.1±2.0	63.5±4.6
	MELM(2022) [38]	30.7±2.2	48.9±2.0	62.7±1.1
	KGPC(2023) [12]	51.0±5.6	67.8±4.2	71.5±1.6
	Gemini-1.0	56.4±1.9	52.3±2.8	–
	Gemini-1.5	62.4±0.3	63.2±0.2	–
	GPT-3.5-turbo	46.9±0.5	42.4±0.6	–
	GPT-3.5-turbo-0125	48.2±0.9	43.3±0.5	–
	Ours	**62.6** $\varvec{\pm }$**11.5**	**70.3** $\varvec{\pm }$**5.7**	**73.9** $\varvec{\pm }$**3.1**
BC5CDR-Disease	TransferBERT(2019) [34]	19.8±6.4	44.3±4.3	54.6±2.5
	Daga(2020) [29]	27.4±3.3	43.3±1.9	52.9±2.4
	NNshot(2020) [35]	36.7±4.3	27.9±4.4	34.0±2.7
	LightNER(2022) [36]	33.5±8.9	56.3±2.8	69.0±2.1
	FFF-NER(2022) [37]	41.6±4.1	47.2±6.2	71.5±2.8
	MELM(2022) [38]	35.9±1.9	48.8±5.5	56.1±3.8
	KGPC(2023) [12]	49.2±7.7	65.5±3.2	67.8±1.0
	Gemini-1.0	57.1±0.2	52.9±0.3	–
	Gemini-1.5	60.8±0.2	59.1±0.5	–
	GPT-3.5-turbo	47.5±0.5	43.4±0.4	–
	GPT-3.5-turbo-0125	49.9±1.1	45.4±0.9	–
	Ours	**71.0** $\varvec{\pm }$**2.4**	**74.9** $\varvec{\pm }$**1.6**	**78.2** $\varvec{\pm }$**1.0**
BioNLP11EPI	TransferBERT(2019) [34]	51.8±1.2	58.6±1.1	59.5±1.4
	Daga(2020) [29]	43.0±13.2	57.7±1.1	61.7±2.1
	NNshot(2020) [35]	34.0±10.3	40.9±1.6	47.0±2.1
	LightNER(2022) [36]	49.6±6.5	53.9±2.0	65.4±2.8
	FFF-NER(2022) [37]	47.7±3.8	53.7±2.0	67.3±2.2
	MELM(2022) [38]	49.2±4.8	54.4±2.6	58.7±1.3
	KGPC(2023) [12]	55.0±10.0	63.8±1.2	**71.6** $\varvec{\pm }$**1.3**
	Gemini-1.0	57.2±0.7	61.0±0.3	–
	Gemini-1.5	**64.3** $\varvec{\pm }$**0.6**	**65.0** $\varvec{\pm }$**0.2**	–
	GPT-3.5-turbo	50.8±0.1	55.0±0.9	–
	GPT-3.5-turbo-0125	53.1±0.8	55.8±0.3	–
	Ours	45.2±6.6	59.8±1.0	66.6±0.6
BioNLP13GE	TransferBERT(2019) [34]	40.0±7.6	54.4±2.5	62.5±1.9
	Daga(2020) [29]	40.4±3.8	61.5±0.8	63.3±1.6
	NNshot(2020) [35]	27.3±5.3	46.6±1.8	50.1±2.3
	LightNER(2022) [36]	42.4±3.9	59.8±4.8	63.2±1.7
	FFF-NER(2022) [37]	49.8±1.6	56.8±2.9	66.7±4.9
	MELM(2022) [38]	42.8±5.8	58.6±2.8	55.4±3.4
	KGPC(2023) [12]	48.4±7.8	71.3±2.6	68.3±2.1
	Gemini-1.0	49.2±3.8	53.9±0.6	–
	Gemini-1.5	**55.8** $\varvec{\pm }$**0.0**	64.0±0.5	–
	GPT-3.5-turbo	40.6±0.9	48.7±0.6	–
	GPT-3.5-turbo-0125	41.2±0.5	53.7±1.0	–
	Ours	44.1±11.7	**66.1** $\varvec{\pm }$**2.0**	**70.3** $\varvec{\pm }$**1.2**

Notably, KGPC, MELM, and DaGa employed different strategies than ours. Our advantage lies in utilizing ChatGPT-based methods, which leverage text generation from LLMs to enhance diversity and authenticity in natural language, simulating real usage patterns. This naturalness and diversity bolster the model’s generalization capabilities, enabling better adaptation to various contexts and text styles. Furthermore, incorporating dynamic convolution enhances feature representation, improving the capture of entity features and contextual information, making our model more effective in few-shot learning scenarios.

For the BIO11EPI dataset, our results show a performance lag compared to KGPC’s. We attribute this difference to KGPC’s approach, which utilizes knowledge graphs and question-answer CL, tailored to the specific features of the BioNLP11EPI dataset, resulting in superior performance. To address this discrepancy, we plan to analyze the unique attributes of the BioNLP11EPI dataset and refine our model to align better with these characteristics. This involves exploring various strategies or feature representation methods to enhance the model’s adaptation to the unique traits of the BioNLP11EPI dataset.

In comparison to LLMs, our model continues to demonstrate praiseworthy results in most few-shot scenarios. Especially on the NCBI and BC5CDR datasets, our model surpasses the top-performing LLMs models with the highest F1 score differences being 7.1 and 5.8, respectively.

In summary, our model achieved SOTA results in three out of the four datasets across various shot settings in the experiments.

### Analysis and discussion

#### Ablation study

In order to validate the effectiveness of our proposed model, we conducted ablation studies on partial modules of the system using the NCBI-Disease and BC5CDR-Disease datasets to observe each module’s impact on the experiments. The results of the ablation studies are presented in Table 2 .

**Table 2:** Ablation study results (F1 score (%)) on NCBI and BC5CDR-Disease datasets

Dataset	Model	5-shot	20-shot	50-shot
NCBI	Ours	**62.6** $\varvec{\pm }$**11.5**	**70.3** $\varvec{\pm }$**5.7**	**73.9** $\varvec{\pm }$**3.1**
	-Multi-scale	31.8±1.6	64.6±7.1	70.5±4.7
	-Dconv	62.5±7.2	69.2±5.0	72.2±3.1
	-Gate	54.9±10.3	65.0±8.5	71.7±2.6
	-Attention	59.7±7.8	66.5±5.5	70.1±1.9
	-DA	34.12±3.6	57.9±11.4	69.3±6.8
BC5CDR-Disease	Ours	**71.0** $\varvec{\pm }$**2.4**	**74.9** $\varvec{\pm }$**1.6**	**78.2** $\varvec{\pm }$**1.0**
	-Multi-scale	62.9±4.0	72.3±1.2	76.2±1.5
	-Dconv	70.3±1.2	73.6±2.8	77.6±0.5
	-Gate	65.9±3.2	73.2±1.6	76.2±2.5
	-Attention	65.4±3.1	73.3±1.8	76.4±0.5
	-DA	37.6±7.8	66.7±3.9	71.9±3.5

**Multi-scale**: Removal of the multi-scale feature extraction module. Results in Table 2 illustrate a significant decrease in performance across all shots on both datasets. Notably, on NCBI with 5-shot, 20-shot, and 50-shot scenarios, the F1 scores decreased by 30.8%, 5.7%, and 3.4%, respectively. The absence of the multi-scale feature extraction module led to a loss in capturing richer semantic information from sentences. Multi-scale features notably benefit scenarios with smaller sample sizes.

**Dconv**: Removal of the dynamic convolution module. In our study, we introduced dynamic convolution modules from the visual domain to further extract local sentence features to enhance the model’s understanding of sentences. Results in Table 2 show that removing the dynamic convolution module resulted in a slight decrease in model performance, indicating a loss of local sentence features. Specifically, on NCBI, the F1 scores decreased by 0.1%, 1.1%, and 1.7% across three shot scenarios.

**Gate**: Removal of the gating mechanism. Without the gating mechanism and employing direct feature addition for fusion, Table 2 indicates a significant decrease in F1 scores on both datasets. On NCBI, the F1 scores decreased by 7.7%, 5.3%, and 2.2% across three shot scenarios. The gating mechanism facilitates selective feature integration, allowing the model to selectively attend to and integrate input features, aiding in extracting relevant information for the task while ignoring less important features. This capability contributes to enhancing the model’s effectiveness and robustness.

**Attention**: Removal of the attention mechanism, employing direct addition for fusion of extracted multi-scale features. Though retaining multi-scale features, the fusion method also plays a crucial role. The absence of the attention mechanism led to a decrease in F1 scores on BC5CDR-Disease dataset by 5.6%, 1.6%, and 1.8% across three shot scenarios. This indicates that guided by the attention mechanism, purposeful fusion of multi-scale features can enhance model performance. These results underscore the significance of each module in contributing to the model’s performance, demonstrating their individual roles in enhancing the model’s effectiveness in handling low-resource BioNER tasks.

**DA**: In addition, we conducted experiments by excluding any methods. The results, as shown in Table 2, indicate a significant decline in model performance in the absence of . This underscores the substantial impact of on model efficacy. It is noteworthy, particularly in the 5-shot setting, that the performance of the model without relying on is not satisfactory, marking a critical aspect for further optimization in our future work.

**Table 3:** Model performance (F1 score (%)) enhanced with UMLS and ChatGPT on NCBI, BC5CDR-Disease, BioNLP11EPI, and Bio13GE datasets

Dataset	DA	5-shot	20-shot	50-shot
NCBI	ChatGPT	**62.6** $\varvec{\pm }$**11.5**	**70.3** $\varvec{\pm }$**5.7**	**73.9** $\varvec{\pm }$**3.1**
	UMLS* [12]	55.7±9.3	65.0±5.2	68.6±3.3
BC5CDR-Disease	ChatGPT	**71.0** $\varvec{\pm }$**2.4**	**74.9** $\varvec{\pm }$**1.6**	**78.2** $\varvec{\pm }$**1.0**
	UMLS* [12]	62.1±6.0	63.7±5.5	67.0±2.3
BioNLP11EPI	ChatGPT	**45.2** $\varvec{\pm }$**6.6**	**59.8** $\varvec{\pm }$**1.0**	**66.6** $\varvec{\pm }$**0.6**
	UMLS* [12]	40.0±9.5	51.3±3.5	58.8±2.3
BioNLP13GE	ChatGPT	**44.1** $\varvec{\pm }$**11.7**	**66.1** $\varvec{\pm }$**2.0**	**70.3** $\varvec{\pm }$**1.2**
	UMLS* [12]	37.4±3.4	48.5±2.7	52.7±1.7

#### Comparison experiment on data augmentation

Due to the limited sample size, employing techniques is a common and effective approach in the domain of FSL to enhance model performance. To explore the impact of different augmentation methods on model performance, we compared two distinct augmentation approaches across four datasets. One approach utilized knowledge-guided UMLS [12] augmentation proposed by Chen et al., while the other was based on the ChatGPT large language model. Detailed experimental results in terms of F1 scores are reported in Table 3. It is noteworthy that in Table 3, entries marked with “*” indicate experiments where we replaced the ChatGPT-based augmentation with the augmentation method proposed by Chen et al.

In Table 3, it’s evident that models augmented using ChatGPT significantly outperform those augmented through the UMLS approach. We attribute this advantage to ChatGPT’s flexibility in providing text with similar semantics but diverse expressions. This flexibility enables ChatGPT to generate more varied and contextually diverse data, thereby enhancing the model’s understanding of different medical domain contexts. Moreover, text generated by ChatGPT better reflects the diversity and authenticity of natural language. Its training on a LLMs allows it to simulate natural language usage more effectively. This naturalness and diversity contribute to improving the model’s generalization ability, enabling it to better adapt to diverse contexts and text styles.In contrast, UMLS-based augmentation may be more constrained by existing terminologies and synonym libraries. This constraint might lead to generated text lacking sufficient diversity and authenticity, thereby limiting the model’s ability to generalize.

#### Comparison experiment on different conv method

To explore deeper into the advantages of the proposed model, we further demonstrate the superiority of the dynamic convolution used in this paper by conducting comparative experiments with static convolution, thereby proving the effectiveness of the convolution selected in this study. As can be seen from Table 4, the performance of dynamic convolution consistently surpasses that of static convolution across all few-shot scenarios in all datasets.

**Table 4:** Model performance (F1 score (%)) enhanced with UMLS and ChatGPT on NCBI, BC5CDR-Disease, BioNLP11EPI, and Bio13GE datasets

Dataset	Conv Method	5-shot	20-shot	50-shot
NCBI	Dynamic Conv	**62.6**	**70.3**	**73.9**
	Static Conv	59.3 (−3.3)	67.7 (−2.6)	70.5 (−3.4)
BC5CDR-Disease	Dynamic Conv	**71.0**	**74.9**	**78.2**
	Static Conv	67.2 (−3.8)	69.4 (−5.5)	73.8 (−4.4)
BioNLP11EPI	Dynamic Conv	**45.2**	**59.8**	**66.6**
	Static Conv	44.6 (−0.6)	55.3 (−4.5)	62.9 (−3.7)
BioNLP13GE	Dynamic Conv	**44.1**	**66.1**	**70.3**
	Static Conv	39.7 (−4.4)	59.6 (−6.5)	65.2 (−5.1)

After replacing the model with static convolution, the performance on the three few-shot scenarios of the NCBI dataset decreased by an average of 3.1; on the BC5CDR dataset, it decreased by an average of 4.6; on the BioNLP11EPI dataset, it decreased by an average of 2.9; and on the BioNLP13GE dataset, it decreased by an average of 5.3. This had a significant impact on each dataset. The experimental results prove the effectiveness of the model proposed in the paper. They also validate the superiority of dynamic convolution in NER tasks.

#### Comparison experiment on data augmentation times

**Fig. 3:**
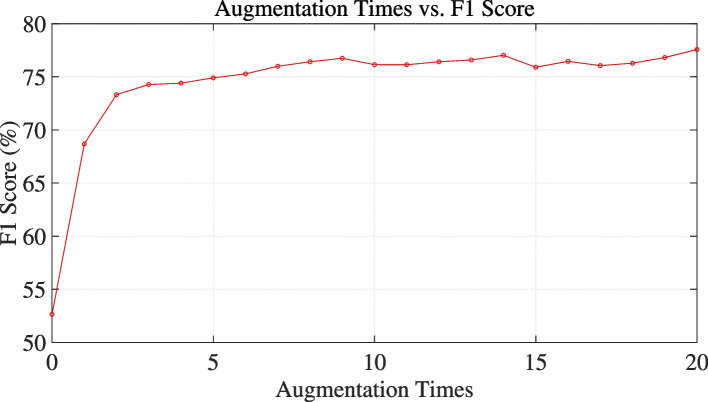
Comparison experiment on data augmentation times

In order to deeply investigate the constraints and efficacy of data augmentation with ChatGPT, this study performed 20 iterations of data augmentation and analyzed the outcomes for augmentation multiples from 1 to 20. From the experimental results shown in the Fig. 3, it can be observed that the model’s performance shows a significant upward trend when augmented five times; beyond five times, the model’s performance stabilizes but still exhibits a slight upward trend overall. Therefore, when using this method, selecting five augmentations is highly appropriate. As the number of augmentations increases, the improvement in model performance becomes minimal, and there is also a possibility of redundancy in the augmented data, as seen when augmenting 15 times, where the model’s performance slightly declines.

#### Visualization

**Fig. 4:**
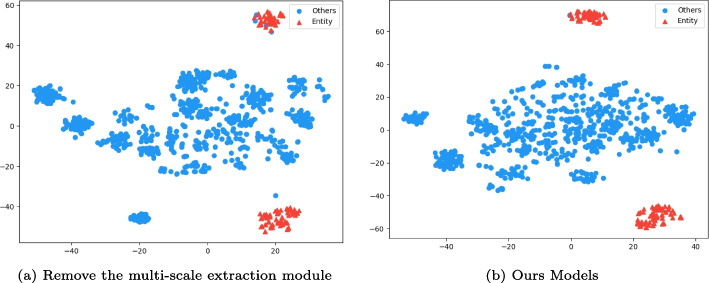
Using t-SNE visualization on the validation set displays the impact of multi-scale features on the model. Entities are indicated by red triangles, while non-entities are represented by blue dots. The model with multi-scale feature extraction shows a more compact distribution

To offer a more visual comparison of the impact of multi-scale feature extraction on the model, we conducted a t-SNE visualization experiment on a randomly selected shot from the NCBI dataset’s validation set, as depicted in Fig. 4. In Fig. 4a, it illustrates the distribution of entities and non-entities after excluding the multi-scale feature extraction module. Figure 4b represents the entity and non-entity distribution on the complete model. Red triangles indicate entities, while blue circles denote non-entities. It’s noticeable that upon incorporating the multi-scale feature extraction module, entities exhibit increased cohesion, with a reduction in the number of non-entity blue circles within the red triangles. The denser distribution of non-entities represented by the blue circles signifies the considerable assistance provided by multi-scale feature extraction in enhancing the model’s performance.

#### Pre-trained language model comparison

**Fig. 5:**
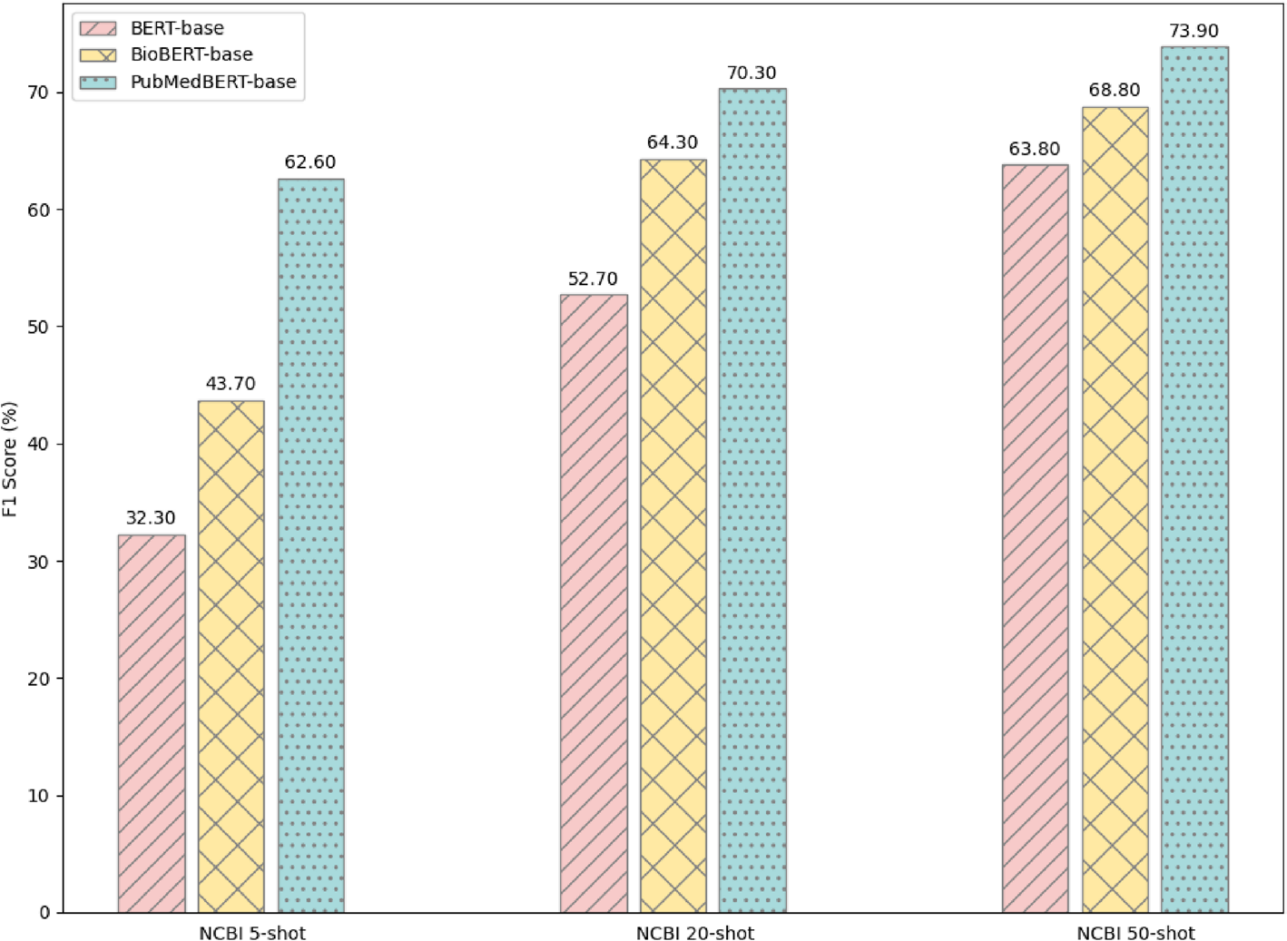
Performance F1 value of different pre-trained language models on NCBI

To explore the impact of different pretrained language models (PLMs) on our experiment, we conducted comparative experiments using three distinct PLMs: BERT [34], BioBERT [39], and PubMedBERT [40]. In order to visually assess the differences, we generated a bar chart Fig. 5 to illustrate the performance variation across these PLMs. Our experiments were conducted on 5-shot, 20-shot, and 50-shot tasks within the NCBI dataset,all three BERT models utilized the base versions. Notably, the general-purpose BERT-base-cased model exhibited marked inferiority compared to the domain-specific BioBERT and PubMedBERT, aligning with our expectations given our utilization of biomedical datasets. Of significance, PubMedBERT outperformed BioBERT, possibly attributed to its pretraining on data closely aligned with biomedical texts from PubMed articles and pertinent literature in the field. In contrast, BioBERT may have undergone pretraining on broader textual domains rather than focusing specifically on biomedical contexts as PubMedBERT did. Consequently, PubMedBERT may have more effectively captured the specific contexts, terminologies, and entity relationships within the biomedical domain, leading to superior performance in BioNER tasks. As a result, PubMedBERT was selected as the final encoder for our experiments.

## Conclusion

In this study, we introduce a model designed to address the challenges of FSL in biomedical NER tasks, aiming to better adapt to realistic scenarios within the biomedical domain. Our model architecture is grounded in multi-scale local feature extraction, dynamic convolution modules, and gate mechanisms. Experimental evaluations were conducted on diverse datasets, including NCBI and BC5CDR-Disease, demonstrating significant performance enhancements across 5-shot, 20-shot, and 50-shot scenarios. Particularly noteworthy is our model’s outstanding increase of up to 20% in the best-case F1 score in the resource-constrained 5-shot scenario, underscoring its effectiveness in low-resource settings. By integrating multiscale information and feature fusion mechanisms, our model design exhibits exceptional performance in the domain of FSL tasks. While some specific datasets exhibit slightly lower performance, we believe this points the way for future refinements in the field. Our research provides an effective solution to the biomedical NER task. We plan to integrate LLMs to explore more challenging zero-shot NER tasks. Specifically, we will leverage LLMs along with advanced prompt engineering and fine-tuning techniques to design a self-correcting framework that optimizes the output of LLMs. This will improve their instruction compliance and answer quality, thereby improving performance in zero-shot scenarios.

## Data Availability

No datasets were generated or analysed during the current study.
